# Transcriptome Analysis of Native Kentucky Bluegrass (*Poa pratensis* L.) in Response to Osmotic Stress

**DOI:** 10.3390/plants12233971

**Published:** 2023-11-25

**Authors:** Jinjing Cheng, Leilei Xiang, Meizhen Yang, Ying Liu, Luyi Pan, Zhenfei Guo, Shaoyun Lu

**Affiliations:** 1 Guangdong Engineering Research Center for Grassland Science, College of Life Sciences, South China Agricultural University, Guangzhou 510642, China; chengjj@stu.scau.edu.cn (J.C.); 20201002007@stu.scau.edu.cn (L.X.); 20191002009@stu.scau.edu.cn (M.Y.); 2 College of Grassland Science, Qinhai University, Xining 210095, China; liuying_yanhong@sina.com; 3 Instrumental Analysis and Research Center, South China Agricultural University, Guangzhou 510642, China; panluyi@scau.edu.cn; 4 College of Grassland Science, Nanjing Agricultural University, Nanjing 210095, China

**Keywords:** Kentucky bluegrass, osmotic stress, transcriptomes analysis, carbohydrate metabolism, polyamine and amino acid metabolism, plant hormone signaling pathway

## Abstract

Kentucky bluegrass (*Poa pratensis* L.) is an important cool season turfgrass species with a high cold tolerance, but it is sensitive to drought. It is valuable for the applications of Kentucky bluegrass to improve its drought tolerance. However, little is known about the underlying drought mechanism. In the present study, transcriptomic profiling in the roots and leaves of the Kentucky bluegrass cultivar ‘Qinghai’, in response to osmotic stress in the form of treatment with 2 h and 50 h of 25% (*v*/*v*) PEG-6000, was analyzed. The results showed that a large number of genes were significantly up-regulated or down-regulated under osmotic stress. The majority of genes were up-regulated in leaves but down-regulated in roots after 2 h and 50 h of osmotic stress, among them were 350 up-regulated DEGs and 20 down-regulated DEGs shared in both leaves and roots. GO and KEGG analysis showed that carbohydrate metabolism, polyamine and amino acid metabolism and the plant hormone signaling pathway were enriched in the leaves and roots of ‘Qinghai’ after osmotic stress. The genes involving in carbohydrate metabolism were up-regulated, and sucrose, trehalose and raffinose levels were consistently increased. The genes involved in polyamine and amino acid metabolism were up-regulated in leaves in response to osmotic stress and several amino acids, such as Glu, Met and Val levels were increased, while the genes involved in photosynthesis, carbon fixation and citrate cycle in leaves were down-regulated. In addition, the genes involved in plant hormone biosynthesis and signal transduction were altered in leaves after osmotic stress. This study provided promising candidate genes for studying drought mechanisms in ‘Qinghai’ and improving the drought tolerance of Kentucky bluegrass and drought-sensitive crops.

## 1. Introduction

Drought is the most common environmental stress that severely restricts plant growth and development [1]. Drought stress alters the expression of thousands of genes that results in biochemical, physiological and morphological changes in plants [2]. Photosynthesis, which is considered to be one of the most crucial biological processes for the survival of plants, is greatly affected by stomatal closure during drought stress [3], and as such, carbon fixation will be hindered [4]. Plant hormones are involved in plant adaptation to drought. Abscisic acid (ABA) is accumulated after plants are exposed to drought, which regulates stomata closure and downstream gene expression through ABA signaling [3]. Cytokinin (CTK) delays leaf senescence under drought stress [4]. Auxin (IAA) and gibberellin (GA) levels are decreased in response to drought, resulting in reduced growth for saving energy [5,6]. Polyamines (PAs) play a certain role in growth and development and in resisting adverse environmental factors. Levels of PAs including putrescine (Put), spermidine (Spd), and spermine (Spm) are increased in plants under drought conditions [7]. PAs regulate the antioxidant defense system to scavenge reactive oxygen species (ROSs) under stress conditions [8]. γ-Aminobutyric acid (GABA), that is produced from polyamine oxidation, is accumulated to protect plants against drought stress by increasing osmolytes and reducing oxidative damage via antioxidants [9]. Soluble sugars are one of the small molecular osmolytes induced by drought stress, among which sucrose is the main soluble sugar. They are largely accumulated under drought stress as a result of starch degradation, while the intermediates in sugar metabolism provide a carbon skeleton for amino acid synthesis [10].

Irrigation is an essential cultivation tool for maintaining turf quality. The turfgrass species or cultivar with increased drought tolerance is always a major issue in urban landscape and sports field applications [11]. Kentucky bluegrass (*Poa pratensis* L.) is one of the most important cool-season type turfgrass species. It has a high cold tolerance but is sensitive to drought. Changes in fatty acid composition and saturation levels and antioxidant enzyme activities are involved in drought tolerance in Kentucky bluegrass [12]. The drought-tolerant cultivar ‘Midnight’ maintains a higher net photosynthetic rate (*P*_n_), and higher activities of ribulose-1,5-bisphosphate carboxylase (Rubisco) and glyceraldehyde phosphate dehydrogenase (GADPH) than the drought-sensitive cultivar ‘Brilliant’ during drought stress [13]. In addition, drought stress-induced injury to Kentucky bluegrass is associated with hormonal alteration, and the plants with higher levels of CTK and IAA and lower levels of ABA have better photosynthetic function and performance under drought stress [14]. Exogenous application of ethephon, silicate and 5-aminolevulinic acid increases drought tolerance in Kentucky bluegrass, with improved photosynthesis and an antioxidant defense system under drought stress [15,16,17]. An RNA-seq analysis of the leaves of the cultivar ‘Midnight II’ in response to PEG-6000 treatment revealed that DEGs were enriched in “plant hormone signal transduction” and the “MAPK signaling pathway”. Some up-regulated DEGs included *PYL*, *JAZ* and *BSK* involved in the hormone signaling transduction of ABA, jasmonic acid (JA) and brassinosteroid (BR) [18]. An RNA-seq approach using three germplasm sources with different drought tolerances identified transcript isoforms exhibiting a shared response of all three germplasm sources to drought stress and transcript isoforms exhibiting a tolerance response, where the more drought-tolerant germplasm sources exhibited higher transcript differences compared to the drought-susceptible cultivar [19].

A native Kentucky bluegrass cultivar named ‘Qinghai’ with extreme cold tolerance was selected from the collections in Dari County, Qinghai Province, which is located in the alpine cold region at an attitude of 4000 m [20]. The molecular responses to drought in ‘Qinghai’ have not been investigated, and yet it is important for breeders to use this special gene resource to improve drought tolerance in Kentucky bluegrass by using modern biotechnological tools. The objective of this study was to investigate transcriptomic responses in ‘Qinghai’ to osmotic stress at early stage of osmotic treatment (2 h to 50 h) and the enriched KEGG pathways. Based on the analysis, some candidate key genes associated with drought tolerance could be selected and identified in the future.

## 2. Results

### 2.1. Global Analysis of Gene Expression Profiles and the Differentially Expressed Genes (DEGs) in Response to Osmotic Stress

Transcriptomic analysis based on deep RNA-seq was performed to understand the global gene expression profiles in ‘Qinghai’ in response to osmotic stress. A total of 18 cDNA samples from leaves and roots were sequenced using the Illumina. A total of 407,753,079 raw reads were obtained (Table S1). After removing the low-quality reads and adaptor sequences, 398,144,703 clean reads were obtained (Table S1). The clean reads were then de novo assembled using Trinity software (2.4.0), and a total of 1,090,844 transcripts were obtained. The average length of the transcripts was 831 bp, and the N50 length was 1049 bp (Table S2). In addition, 569,270 unigenes were obtained with an average length of 737 bp, and the N50 length was 870 bp (Table S2). Among them 365,785 (64.25%) unigenes could be matched to at least one database. 271,475 (47.68%), 116, 438 (20.45%), 121,782 (21.39%), 204,584 (35.93%), 248,701 (43.68%) and 103,921 (18.25%) unigenes were matched to the Non-Redundant Protein Sequence Database (Nr), NCBI nucleotide sequences (Nt), the Kyoto Encyclopedia of Genes and Genomes (KO), Swiss-prot, Pfam, Gene Ontology (GO) and Clusters of Orthologous Groups of proteins (KOG), respectively, while only 23,636 (4.15%) unigenes could be matched to all databases (Table S3).

**Figure 1:**
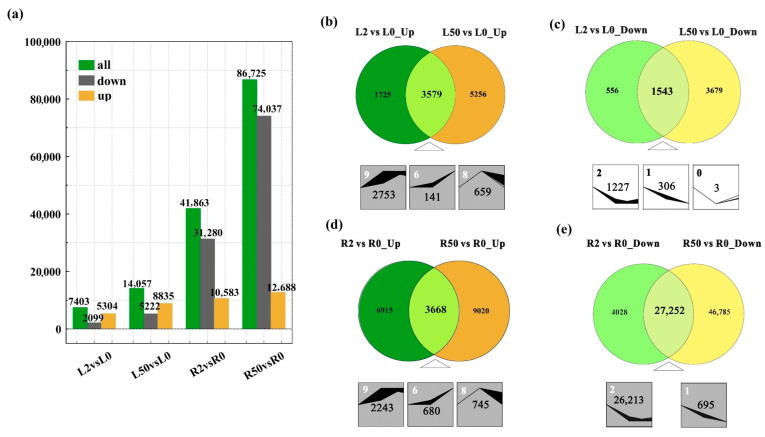
Global analysis of differentially expressed genes (DEGs) in leaves and roots after osmotic stress. (**a**) The number of DEGs in ‘Qinghai’ under osmotic stress; (**b**–**e**) the comparative analysis and trend profiles of DEGs in leaves and roots of ‘Qinghai’ after osmotic stress. L0, L2 and L50 indicate the genes in leaves at 0 h, 2 h or 50 h after osmotic stress. R0, R2 and R50 indicate the genes in roots at 0 h, 2 h or 50 h after osmotic stress. Expression profiles were ordered by the number of differentially expressed genes. Blocks indicate significant enrichment trends (*p* ≤ 0.05). The top-left number in blocks represents the trend ID, and the different trend ID indicates different expression trends. The middle number in blocks represents the number of genes.

A total of 7403 DEGs (5304 up-regulated and 2099 down-regulated) and 14,057 DEGs (8835 up-regulated and 5222 down-regulated) were obtained from leaves after 2 h and 50 h of osmotic treatment, respectively, while 41,863 DEGs (10,583 up-regulated, 31,280 down-regulated) and 86,725 DEGs (12,688 up-regulated, 74,037 down-regulated) were obtained from roots after 2 h and 50 h, respectively (Figure 1a). The data indicated that more genes were altered by osmotic treatment in roots than in leaves, and the majority of genes were up-regulated in leaves but down-regulated in roots. The Venn diagram shows that 3579 DEGs among the up-regulated genes in leaves were shared at 2 h and 50 h after osmotic treatment, and they showed three trend profiles, including 2753 DEGs in profile 9, 141 DEGs in profile 6 and 659 DEGs in profile 8 (Figure 1b). Among the down-regulated genes in leaves, 1543 DEGs were shared at 2 h and 50 h after osmotic treatment, and they were shown in profile 2 (1227 DEGs), profile 1 (306 DEGs) and profile 0 (3 DEGs) (Figure 1c). Among the up-regulated genes in roots, 3668 DEGs were shared at 2 h and 50 h after osmotic treatment, and they showed patterns in profile 9 (2243 DEGs), profile 6 (680 DEGs) and profile 8 (745 DEGs), respectively (Figure 1d). Among the down-regulated genes in roots 27,252 DEGs were shared at 2 h and 50 h after osmotic treatment, and they showed patterns in profile 2 (26,213 DEGs) and profile 1 (695 DEGs) (Figure 1e).

### 2.2. Analysis of Gene Ontology (GO) and KEGG Pathway Enrichment of DEGs in Leaves

**Figure 2:**
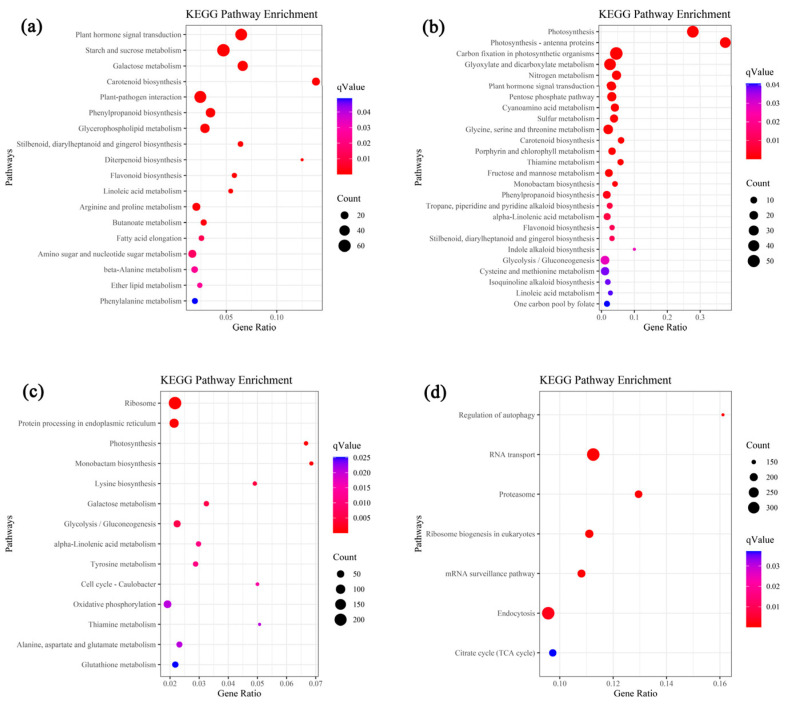
KEGG enrichment analysis of the up- and down-regulated DEGs in leaves and roots after osmotic stress. (**a**) The enriched KEGG pathways of the up-regulated DEGs in leaves shared at 2 h and 50 h after osmotic stress. (**b**) The enriched KEGG pathways of the down-regulated DEGs in leaves shared at 2 h and 50 h after osmotic stress. (**c**) The enriched KEGG pathways of the up-regulated DEGs in roots shared at 2 h and 50 h after osmotic stress. (**d**) The enriched KEGG pathways of the down-regulated DEGs in roots shared at 2 h and 50 h after osmotic stress. The *q*-value is represented by colors from blue to red. The size of the dark dots reflects the number of DEGs involved in each metabolism pathway.

The up-regulated DEGs (3579) shared at 2 h and 50 h after osmotic stress in leaves were analyzed using GO and KEGG enrichment. Based on *q*-value < 0.05, 208 GO terms and 18 KEGG pathways were enriched. The top 30 GO terms including 20 terms in “biological process” and ten terms in “molecular function” are listed in Figure S1a. The enriched pathways included “plant hormone signal transduction” (55 genes), “starch and sucrose metabolism” (63 genes), “galactose metabolism” (39 genes), “carotenoid biosynthesis” (21 genes), “plant-pathogen interaction” (56 genes), “phenylpropanoid biosynthesis” (32 genes), “glycerophospholipid metabolism” (31 genes), “stilbenoid, diarylheptanoid and gingerol biosynthesis” (ten genes), “diterpenoid biosynthesis” (six genes), “flavonoid biosynthesis” (nine genes), “linoleic acid metabolism” (eight genes), “arginine and proline metabolism” (21 genes), “butanoate metabolism” (12 genes), “fatty acid elongation” (ten genes), “amino sugar and nucleotide sugar metabolism” (21 genes), “beta-alanine metabolism” (14 genes), “ether lipid metabolism” (nine genes), “phenylalanine metabolism” (11 genes) (Figure 2a).

The down-regulated DEGs (1543) shared at 2 h and 50 h after osmotic stress in leaves were analyzed using GO and KEGG enrichment. Based on *q*-value < 0.05, 286 GO terms and 26 KEGG pathways were enriched. The top 30 GO terms are listed in Figure S1b, including “biological process” (11 terms), “cellular component” (13 terms) and “molecular function” (6 terms). The enriched pathways included “photosynthesis” (46 genes), “photosynthesis-antenna proteins” (37 genes), “carbon fixation in photosynthetic organisms” (55 genes), “glyoxylate and dicarboxylate metabolism” (46 genes), “nitrogen metabolism” (24 genes), “plant hormone signal transduction” (26 genes), “pentose phosphate pathway” (24 genes), “cyanoamino acid metabolism” (17 genes), “sulfur metabolism” (17 genes), “glycine, serine and threonine metabolism” (27 genes), “carotenoid biosynthesis” (9 genes), “porphyrin and chlorophyll metabolism” (13 genes), “thiamine metabolism” (8 genes), “fructose and mannose metabolism” (15 genes), “monobactam biosynthesis” (6 genes), “phenylpropanoid biosynthesis” (15 genes), “tropane, piperidine and pyridine alkaloid biosynthesis” (seven genes), “alpha-Linolenic acid metabolism” (11 genes), “flavonoid biosynthesis” (5 genes), “stilbenoid, diarylheptanoid and gingerol biosynthesis” (five genes), “indole alkaloid biosynthesis” (two genes), “glycolysis/gluconeogenesis” (21 genes), “cysteine and methionine metabolism” (18 genes), “isoquinoline alkaloid biosynthesis” (six genes), “linoleic acid metabolism” (four genes) and “one carbon pool by folate” (seven genes) (Figure 2b).

### 2.3. Gene Ontology (GO) and KEGG Pathway Enrichment of DEGs in Roots

The up-regulated DEGs (3668) shared at 2 h and 50 h after osmotic stress in roots were analyzed using GO and KEGG enrichment. Based on *q*-value < 0.05, 401 GO terms and 14 KEGG pathways were enriched. The top 30 GO terms included “biological process” (23 terms), “cellular component” (two terms) and “molecular function” (five terms, Figure S1c). The enriched KEGG pathways included “ribosome” (221 genes), “protein processing in endoplasmic reticulum” (95 genes), “photosynthesis” (11 genes), “monobactam biosynthesis” (ten genes), “lysine biosynthesis” (11 genes), “galactose metabolism” (19 genes), “glycolysis/Gluconeogenesis” (44 genes), “alpha-linolenic acid metabolism” (19 genes), “tyrosine metabolism” (20 genes), “cell cycle-caulobacter” (eight genes), “oxidative phosphorylation” (58 genes), “thiamine metabolism” (seven genes), “alanine, aspartate and glutamate metabolism” (28 genes) and “glutathione metabolism” (32 genes, Figure 2c).

Analysis of GO and KEGG enrichment of the down-regulated DEGs (27252) shared at 2 h and 50 h after osmotic stress in roots showed that, based on *q*-value < 0.05, 899 GO terms and seven KEGG pathways were enriched. The top 30 GO terms included “biological process” (including ten terms), “cellular component” (including six terms) and “molecular function” (including 14 terms, Figure S1d). The enriched KEGG pathways included “regulation of autophagy” (146 genes), “RNA transport” (343 genes), “proteasome” (190 genes), “ribosome biogenesis in eukaryotes” (205 genes), “mRNA surveillance pathway” (197 genes), “endocytosis” (340 genes) and “citrate cycle (TCA cycle)” (185 genes) (Figure 2d).

### 2.4. DEGs Joint Analysis of the Up-Regulated and Down-Regulated in Leaves and Roots

**Figure 3:**
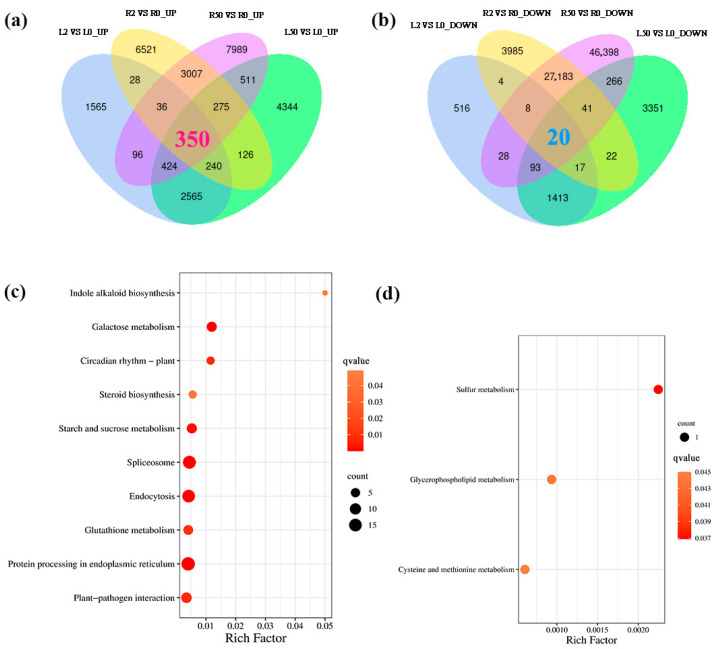
Comprehensive analysis of the DEGs shared in both leaves and roots. (**a**) The number of up-regulated DEGs shared in both leaves and roots after 2 h and 50 h of osmotic stress. (**b**) The number of down-regulated DEGs shared in both leaves and roots after 2 h and 50 h of osmotic stress. (**c**) The enriched KEGG pathways of the up-regulated DEGs shared in both leaves and roots after 2 h and 50 h of osmotic stress. (**d**) The enriched KEGG pathways of the down-regulated DEGs shared in both leaves and roots after 2 h and 50 h of osmotic stress. The red color digit in venn (**a**) represents the number of up-regulated DEGs share in both leaves and roots, the blue color digit in venn (**b**) represents the number of down-regulated DEGs in both leaves and roots. The *q*-value is represented by red color from light red to dark red. The size of the dark dots reflects the number of DEGs involved in each metabolism pathway.

All the up-regulated and down-regulated DEGs were analyzed using a Venn diagram. The results showed that 350 up-regulated DEGs were shared in both leaves and roots at 2 h and 50 h after osmotic treatment (Figure 3a), while 20 down-regulated DEGs were shared (Figure 3b). The up-regulated DEGs were mapped to 10 pathways (Figure 3c), including “protein processing in endoplasmic reticulum” (18 genes), “spliceosome” (16 genes), “galactose metabolism” (seven genes), “endocytosis” (15 genes), “starch and sucrose metabolism” (seven genes), “plant-pathogen interaction” (eight genes), “circadian rhythm-plant” (three genes), “glutathione metabolism” (six genes), “steroid biosynthesis” (three genes), and “indole alkaloid biosynthesis” (one gene) (Figure 3c). Three genes among twenty down-regulated DEGs could be annotated, and they were mapped to “sulfur metabolism”), “glycerophospholipid metabolism”) and “cysteine and methionine metabolism” (Figure 3d).

### 2.5. The Genes Involving in Carbohydrate Metabolism Were Up-Regulated in Leaves and Roots in Response to Osmotic Stress

**Figure 4:**
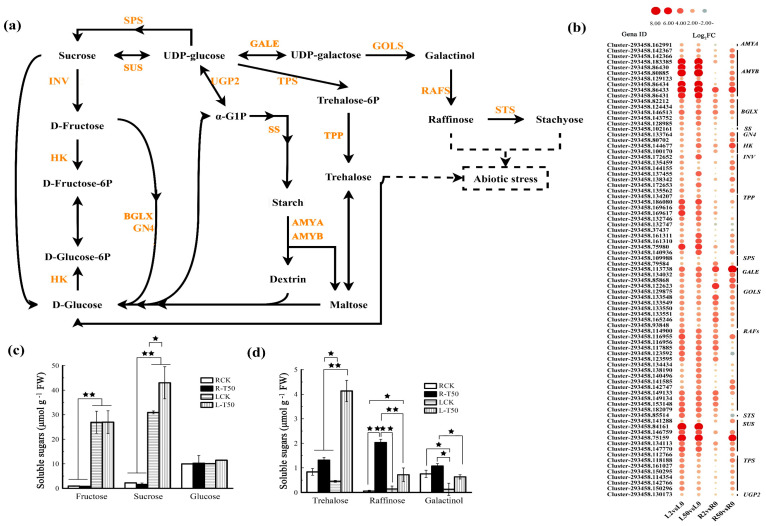
Analysis of DEGs involved in carbohydrate metabolism and soluble sugars in leaves and roots in response to osmotic stress. (**a**) Major metabolism pathway and key enzymes of carbohydrate metabolism. (**b**) The expression patterns of DEGs involved in carbohydrate metabolism. (**c**,**d**) Soluble sugar levels in leaves and roots in response to 50 h of osmotic stress. The dashed lines in pathway represent participation, the dashed boxes represent pathway, the solid lines in pathway represents synthesis. The color spectrum of heat map ranging from blue to red represents the log_2_ FC from low to high, |Log_2_FC| ≥ 1. The soluble sugars were measured after 50 h of treatment with 25% PEG. RCK and LCK indicate the control roots and leaves, respectively, while R-T50 and L-T50 indicate the samples of roots and leaves after 50 h of treatment with 25% PEG. All data are presented as means ± SE from three independent experiments. The asterisks * and ** indicate significant difference at *p* < 0.05 and 0.01, respectively.

Plants accumulate sugars under drought stress. The up-regulated DEGs clustered the in sucrose and starch metabolism pathway combined with those in raffinose, trehalose and stachyose biosynthesis were further analyzed. The metabolic pathway and the key enzymes are shown in Figure 4a. The DEGs included *sucrose-phosphate synthase* (*SPS*, two genes), *sucrose synthase* (*SuSase*, six genes), *β-fructofuranosidase* (*INV*, seven genes) in sucrose biosynthesis and metabolism, *hexokinase* (*HK*, two genes), *glucan endo-1*,*3-beta-glucosidase 4* (*GN4*, two genes), *β-glucosidase* (*BGLX*, five genes) for fructose-6-phosphate and glucose-6-phosphate biosynthesis. In addition, *trehalose 6-phosphate synthase* (*TPS*) and *trehalose 6-phosphate phosphatase* (*TPP*) for trehalose biosynthesis, *inositol 3-α-galactosyltransferase* (*GOLS*, eight genes), *raffinose synthases* (*RAFS*, fifteen genes) and *stachyose synthase* (*STS*, one gene) for raffinose biosynthesis were up-regulated in leaves and roots after osmotic stress. *α-Amylase* (*AMYA*) and *β-amylase* (*BMYB*, nine genes) for starch degradation and *UTP-glucose-1-phosphate uridylyltransferase* (*UGP2*), *starch synthase* (*SSS*) and *UDP-glucose 4-epimerase* (*GALE*) were also up-regulated (Figure 4b).

Soluble sugars in leaves and roots in response to osmotic stress were measured. Fructose and sucrose levels were higher in leaves than in roots (Figure 4c), while trehalose and galactinol levels were higher in roots than in leaves under control condition (Figure 4d). The sucrose level was increased in leaves but not in roots after osmotic stress, while fructose and glucose levels were not altered in either leaves or roots (Figure 4c). Trehalose and raffinose levels were increased in both leaves and roots, while the galactinol level was increased in leaves but not in roots after osmotic stress (Figure 4d).

### 2.6. The Genes Involving in Polyamine and Amino Acid Biosynthesis and Metabolism Were Up-Regulated in Leaves in Response to Osmotic Stress

**Figure 5:**
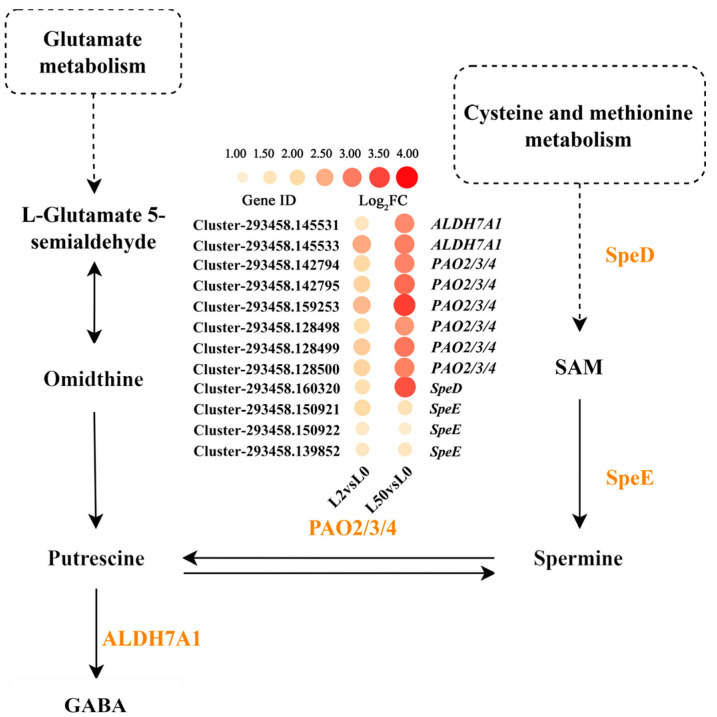
Analysis of DEGs involved in polyamine biosynthesis and metabolism under osmotic stress. The color spectrum ranging from yellow to red represents log_2_FC values from low to high, |Log_2_FC| ≥ 1. The dashed lines represent source, the dashed boxes represent pathway, the solid lines represent synthesis.

*S*-adenosylmethionine decarboxylase (SpeD) and spermidine synthase (SpeE) are key enzymes for polyamine biosynthesis, while polyamine oxidase and aldehyde dehydrogenase (ALDH7A1) catalyze oxidation of polyamines to produce α-aminobutyricacid (GABA). *SpeD*, *SpeE* (three genes), *PAO2*, *3*, *4* (six genes) and *ALDH7A1* (two genes) were up-regulated after 2 h and 50 h of osmotic stress (Figure 5), indicating that polyamine biosynthesis and metabolism were involved in the response to osmotic stress.

**Figure 6:**
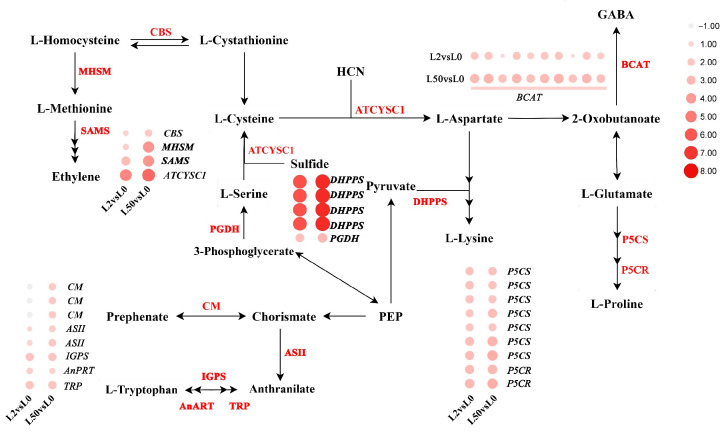
Analysis of DEGs involved in amino acid biosynthesis and metabolism under osmotic stress. Red color represents up-regulated genes. The color spectrum ranging from white to red represents log_2_FC values from low to high, |Log2FC| ≥ 1.

Thirty-one genes involved in amino acid biosynthesis and metabolism were up-regulated in leaves in response to osmotic stress. *δ-1-pyrroline-5-carboxylate synthetase* (*P5CS*, seven genes) and *pyrroline-5-carboxylate reductase* (*P5CR*, two genes) involved in proline biosynthesis were up-regulated after 2 h and 50 h of osmotic stress. *Branched-chain amino acid aminotransferase* (*BCAT*, ten genes) involved in GABA biosynthesis, *chorismate mutase* (*CM*, three genes), *anthranilate synthase component II* (*ASII*, two genes), *indole-3-glycerol phosphate synthase* (*IGPS*), *anthranilate phosphoribosyltransferase* (*AnPRT*) and *phosphoribosylanthranilate isomerase* (*TRP*) involved in tryptophan biosynthesis, *4-hydroxy-tetrahydrodipicolinate synthase* (*DHDPS*, four genes) involved in lysine biosynthesis, *D-3-phosphoglycerate dehydrogenase* (*PGDH*), *L-3-cyanoalanine synthase/cysteine synthase* (*ATCYSC1*) and *cystathionine β-synthase* (*CBS*) involved in cysteine biosynthesis, *5-methyltetrahydropteroyltriglutamate-homocysteine methyltransferase* (*MHSM*) and *S-adenosylmethionine synthetase* (*SAMS*) involved in methionine biosynthesis were up-regulated after 50 h of osmotic stress (Figure 6).

Free amino acids in leaves were detected in response to osmotic stress. Except Cys, Orn, Asp, Ser, Lys, Tyr and Ile, the levels of Glu, Gly, Met, Thr, Ala, Arg, His, Trp, Leu, Pro, Phe and Val in leaves were increased after 50 h of osmotic stress. The Glu, Met and Val were maintained at high levels under both control and osmotic stress conditions and were increased by osmotic stress. Pro, Val and Phe levels showed approximately 20, 36, and 33-fold increases after osmotic stress, respectively (Table 1). In addition, some of the amino acid derivatives, except Tau, Cysthi, β-AiBA and 1-Mehis, were significantly increased after osmotic stress, among them, the GABA level was increased by 43.6-fold (Table 1).

### 2.7. The DEGs Involving in Photosynthesis and Carbon Fixation in Leaves Were Down-Regulated in Response to Osmotic Stress

**Figure 7:**
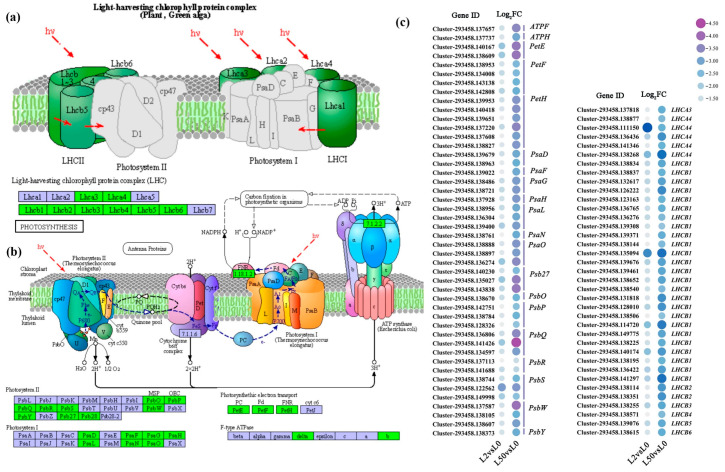
Analysis of DEGs involved in photosynthesis pathway in leaves. (**a**,**b**) Major pathway of photosynthesis and key antenna protein. (**c**) The expression patterns of DEGs involved in photosynthesis pathway. The green rectangles in pathway represent down-regulated DEGs, while the blue rectangles in pathway represent insignificant changes genes. The color spectrum of heat map represents log_2_FC values from high to low, |Log_2_FC| ≥ 1. Schemes were retrieved from KEGG (ko00195, ko00196). Shapes and arrows follow the KEGG representation standards (https://www.kegg.jp/kegg/, accessed on 13 November 2023), except for color codes.

Among the down-regulated DEGs in leaves, eighty-four genes were clustered in antenna protein (Figure 7a) and photosynthesis (Figure 7b) pathways. They included photosystem I (19 genes), photosystem II (13 genes), photosynthesis electron transport (12 genes), F-type ATPase (two genes) and the light-harvesting chlorophyll protein complex (38 genes), which were down-regulated after 2 h and 50 h of osmotic stress (Figure 7c).

**Figure 8:**
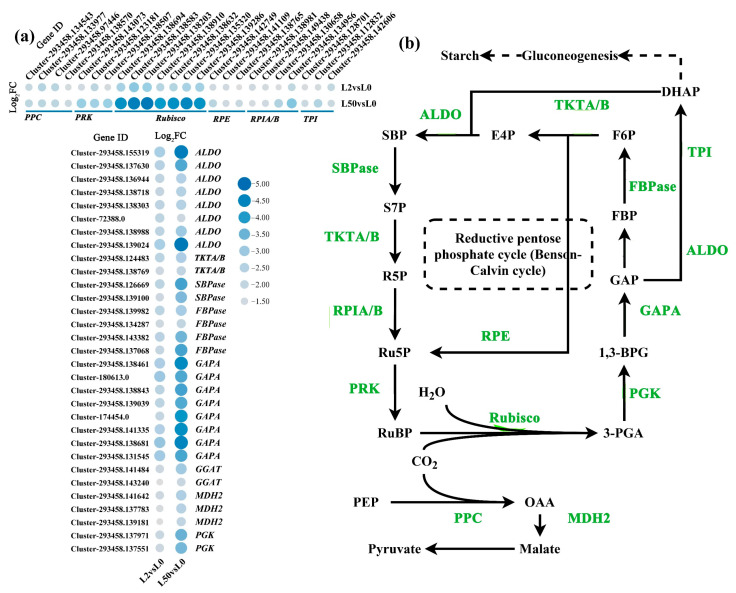
Analysis of DEGs involved in carbon fixation under osmotic stress. (**a**) The expression patterns of DEGs involved in carbon fixation; (**b**) major metabolism pathway and key enzymes of carbon fixation. The green digits represent down-regulated DEGs. Schemes were retrieved from KEGG (ko00710). The dashed lines represent participation, the dashed boxes represent pathway, the solid lines represent synthesis. The color spectrum of heat map ranging from white to blue represents Log_2_FC values from high to low, |Log_2_FC| ≥ 1.

The down-regulated DEGs in glyoxylate and dicarboxylate metabolism pathway were further analyzed. The pathway is shown in Figure 8b, and *ribulose-bisphosphate carboxylase small chain* (*Rubisco*) (seven genes), *phosphoglycerate kinase* (*PGK*) (two genes), *glyceraldehyde-3-phosphate dehydrogenase* (*GAPA*) (eight genes), *fructose-bisphosphate aldolase class I* (*ALDO*) (eight genes), *fructose-1*,*6-bisphosphatase I* (*FBPase*) (four genes) and *sedoheptulose-1*,*7-bisphosphatase* (*SBPase*) (two genes) were down-regulated after 2 h and 50 h of osmotic stress (Figure 8b).

### 2.8. The Expression of Genes Involving in Plant Hormone Biosynthesis and Signal Transduction Were Altered in Leaves in Response to Osmotic Stress

**Figure 9:**
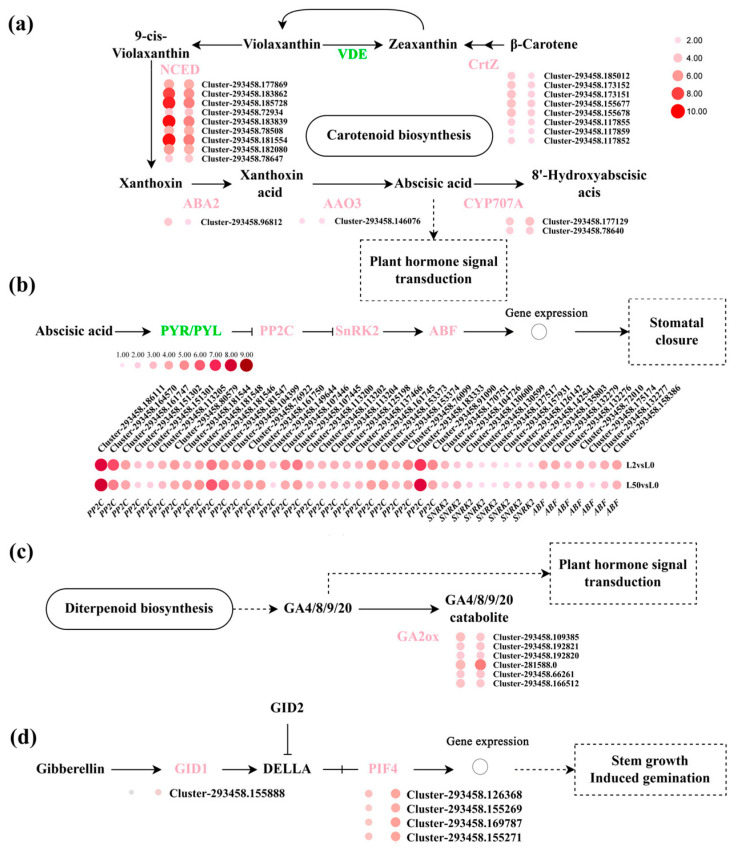
Analysis of DEGs involved in ABA and GA biosynthesis and signal transduction in leaves under osmotic stress. (**a**,**b**) The key enzymes and expression patterns of DGEs in the ABA biosynthesis and signal transduction. (**c**,**d**) The key enzymes and expression patterns of DGEs in the GA biosynthesis and signal transduction. The green-labeled genes represent down-regulated DGEs, while the pink-labeled genes represent up-regulated DGEs. The color spectrum of heat map ranging from white to red represents Log_2_FC values from low to high, |Log_2_FC| ≥ 1. Schemes were adapted from KEGG (ko00906, ko00904, ko04075). The dashed lines represent participation, the dashed boxes represent pathway, the solid lines in (**a**,**c**) represent synthesis, the solid lines in (**b**,**d**) represent signal transduction.

ABA is accumulated under osmotic stress to improve osmotic tolerance. Sixteen DEGs in the ABA biosynthesis pathway were up-regulated after osmotic stress, including three *beta-carotene 3-hydroxylase* (*CrtZ*), nine *9-cis-epoxycarotenoid dioxygenase* (*NCED*), one *xanthoxin dehydrogenase* (*ABA2*) and *abscisic-aldehyde oxidase 3* (*AAO3*), and two *abscisic acid 8’-hydroxylase* (*CYP707A*), but *Violaxanthin deepoxidase* (*VDE*) was down-regulated (Figure 9a). The altered expression of the above genes was consistent with ABA accumulation in plants under osmotic stress. In addition, forty-three genes involved in ABA signal transduction were up-regulated, including eight *ABA-dependent kinases SNF1-regulated protein kinase 2* (*SnRK2s*), twenty-eight *ABA negative regulated genes protein phosphatase 2C* (*PP2C*) and seven *ABA responsive element binding factor* (*ABF*), while four ABA receptor encoding genes *Pyrabactin Resistance 1-like* (*PYL*) were down-regulated (Figure 9b).

Seven genes involved in gibberellins biosynthesis and the signal transduction pathway were altered after drought stress, including six *gibberellin 2-oxidase* (*GA2ox*) genes which were down-regulated and *gibberellin receptor* (*GID1*), *phytochrome-interacting factor 4* (*PIF*) which were up-regulated (Figure 9c,d). Twelve DEGs involved in the auxin signal transduction pathway were down-regulated, including three *auxin influx carriers* (*AUX1*), three *auxin-responsive proteins* (*Aux/IAA*) and two s*mall auxins up RNAs* (*SAUR*) (Figure S2).

### 2.9. The Genes Involving in Citrate Cycle in Roots Were Down-Regulated in Response to Osmotic Stress

**Figure 10:**
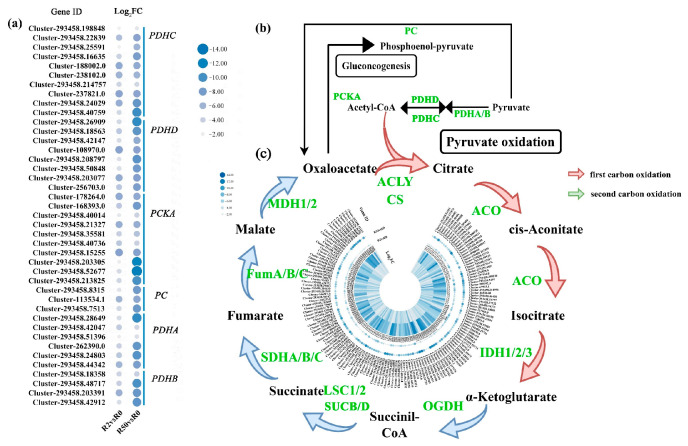
Analysis of DEGs involved in citrate cycle pathway in roots under osmotic stress. (**a**) The expression patterns of the DEGs involved in pyruvate oxidation and gluconeogenesis. (**b**) Major pathway and key enzymes of tyrosine biosynthesis and citrate cycle. (**c**) The key enzymes and expression patterns of the DEGs in TCA. The green-labeled genes represent down-regulated genes. The color spectrum of heat map ranging from white to blue represents log_2_FC values from high to low, |Log_2_FC| ≥ 1.

The TCA cycle is an important aerobic pathway for the oxidation of carbohydrates and fatty acids. The cycle starts with acetyl-CoA and goes back to oxaloacetate at the end of the cycle. *Pyruvate dehydrogenase E1 component alpha subunit* (*PDHA/B/C/D*, 20 genes) expression was down-regulated. *Phosphoenolpyruvate carboxykinase ATP* (*PCKA*, ten genes) in the gluconeogenesis pathway was dramatically down-regulated (Figure 10a,b). A large number of DEGs involving in citrate cycle were down-regulated (Figure 10b,c), including *ATP citrate lyase* (*ACLY*, 11 genes), *citrate synthase* (*CS*, 14 genes), *aconitate hydratase* (*ACO*, 12 genes) and *isocitrate dehydrogenase* (*IDH1/3*, 26 genes) for the first carbon oxidation, and *2-oxoglutarate dehydrogenase E1 component* (*OGDH*, 14 genes), *2-oxoglutarate dehydrogenase E2 component* (*SUCB*, six genes), *succinyl-CoA synthetase alpha subunit* (*LSC1/2*, *19* genes), *succinate dehydrogenase* (*ubiquinone*) *flavoprotein subunit* (*SDHA/B/C*, 14 genes), *class II fumarate hydratase* (*FumA/B/C*, eight genes), *malate dehydrogenases* (*MDH1*, 20 genes).

### 2.10. Verification of Several Differentially Expressed Genes in Resposne to Osmotic Stress

**Figure 11:**
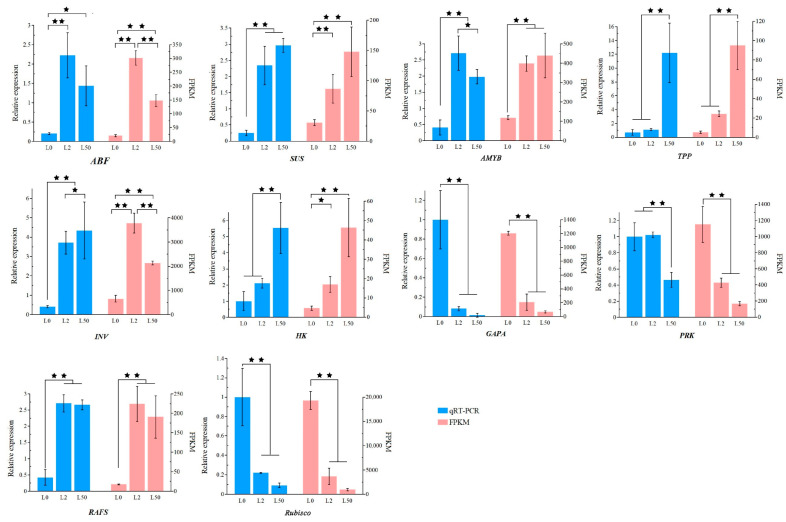
Validation of ten DEGs profiles in leaves using qRT-PCR. All data are presented as means ± SE from three independent experimental replicates. The asterisks * and ** above the column indicate significant difference at *p* < 0.05 and 0.01, respectively.

**Figure 12:**
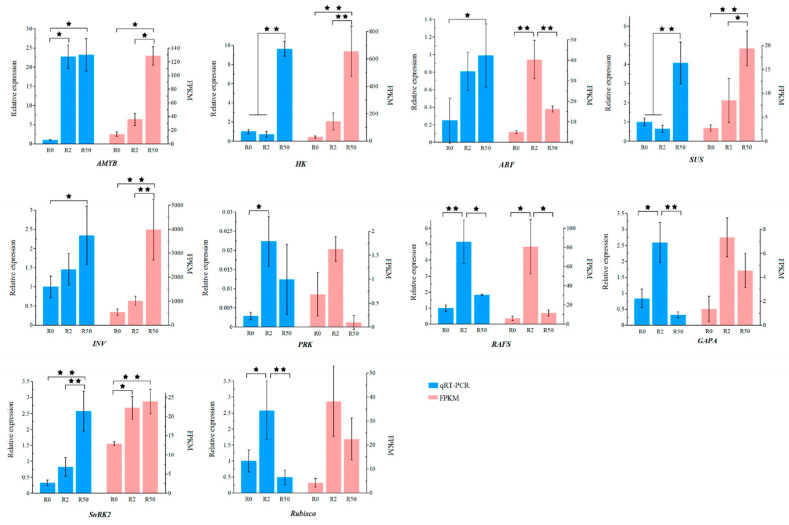
Validation of the ten DEG profiles in roots by qRT-PCR. All data are presented as means± SE from three independent experimental replicates. The asterisks * and ** above the column indicate significant difference at *p* < 0.05 and 0.01, respectively.

Ten DEGs in leaves and roots were randomly selected for validation with qRT-PCR (Figure 11 and Figure 12). Ten DEGs were differentially expressed among roots and leaves after osmotic stress. Among them, *ABF*, *SUS*, *AMYB*, *INV*, *HK*, *TPP* and *RAFS* expression was up-regulated, while *GAPA*, *PRK* and *Rubisco* expression was down-regulated in leaves after osmotic stress for 2 h and 50 h. *AMYB*, *INV*, *SUS*, *GAPA*, *SnRK2*, *ABF*, *PRK*, *RAFS*, *Rubisco* and *HK* expression in roots was significantly up-regulated after osmotic stress. The relative expression of these genes in leaves or roots were consistent with the RNA-seq results.

## 3. Discussion

Plants respond to osmotic stress by changing their morphology and physical and biochemical properties, resulting from alterations in the expression of numerous genes. The transcriptomic profiling of ‘Qinghai’ in response to osmotic stress was analyzed in the present study. The results showed that 3579 genes were continuously up-regulated and 1543 genes were continuously down-regulated in leaves after 2 h and 50 h of osmotic stress, while 3668 genes were continuously up-regulated and 27,252 genes were continuously down-regulated in roots after 2 h and 50 h of osmotic stress. Some differentially enriched pathways were obtained in leaves and roots of ‘Qinghai’ after osmotic stress. Consistent with work on Kentucky bluegrass [19], the stress-related pathways, such as carbohydrate metabolism, polyamine and amino acid metabolism and plant hormone signaling pathway, were also differentially enriched in our research under osmotic stress. Through comparative analysis, 350 up-regulated genes and 20 down-regulated genes shared in both leaves and roots after 2 h and 50 h of osmotic stress were identified. Most of these DEGs are involved in some key biological processes, suggesting these DEGs are crucial in ‘Qinghai’ coping with osmotic stress.

The down-regulated genes in leaves were enriched in the Calvin–Benson cycle, photorespiration, the photosynthetic electron transport chain and antenna proteins. Photosynthesis is the first process affected by drought stress, and photosynthetic proteins have been reported to be the most affected proteins under osmotic stress [21]. The concentration of PSII and PSI proteins as well as *Lhcb* have been shown to decrease in water stress [22]. It is likely that a large number of photosynthesis genes were down-regulated expression, suggesting photosynthesis was severely restricted under osmotic stress. In addition, *ribulose-bisphosphate carboxylase small chain* (*Rubisco*), *phosphoglycerate kinase* (*PGK*), *glyceraldehyde-3-phosphate dehydrogenase* (*GAPA*), *fructose-bisphosphate aldolase class I* (*ALDO*), *fructose-1*,*6-bisphosphatase I* (*FBPase*) and *sedoheptulose-1*,*7-bisphosphatase* (*SBPase*) in carbon fixation were also down-regulated, and carbohydrate synthesis was reduced in ‘Qinghai’ under osmotic stress.

The TCA cycle is not only a bridge connecting carbohydrate, amino acid, lipid and protein metabolism, but also an engine to generate energy and reduce the power needed to drive metabolism [23]. The TCA cycle is also one of the most important protection systems for plants under abiotic stress [24]. Altered levels of L-asparagine and citric acid in the TCA cycle is associated with the difference in drought resistance among the soybeans [25]. Drought-resistant sorghum coped with drought stress through promoting the TCA cycle to improve sphingolipid biosynthesis [26]. In our analysis, the expression of *CS*, *ACO*, *IDH*, *OGDH*, *SUC*, *SDH*, *FUM* and *MDH* in the TCA pathway were down-regulated, suggesting that the TCA cycle in ‘Qinghai’ was inhibited under osmotic stress.

Carbohydrate content will directly affect many physiological processes in plants, such as photosynthesis and the glycolytic pathway [27]. Starch biosynthesis and accumulation were reported to be significantly reduced after drought stress [10]. *α-Amylase* (*AMYA*) and *β-amylase*, used for starch metabolism, were up-regulated. The degradation of starch will provide a substrate for the synthesis of other soluble sugars in response to osmotic stress. High levels of soluble sugar could improve plant resistance. SUS and SPS are key enzymes in the sucrose biosynthesis pathway, and previous research showed that SPS activity increased in wheat under drought stress [28]. INV and HK function promote the hydrolysis of sucrose into glucose. The key genes, *GOLS*, *RAF*s, *STS* and *TPS,* were involve in the trehalose and raffinose biosynthesis and were significantly up-regulated in *C. pilosula* roots after drought stress [29]. The alteration of the expression of key genes in sugar pathways are closely related to plant drought tolerance. These DEGs involved in sucrose, trehalose and raffinose biosynthesis and metabolism were up-regulated in leaves and roots of ‘Qinghai’, which was consistent with the increased sucrose, trehalose and raffinose concentrations after osmotic stress, while fructose and glucose levels were not altered in either leaves or roots (Figure 2c,d). In addition, the concentration of trehalose was dramatically increased in leaves of ‘Qinghai’ compared with the control after osmotic stress due to the constant expression of the active *TPS*s and *TPP*s in leaves, while raffinose accumulates mainly due to the high expression of *RAFS* in roots. The results were similar to many previous studies showing that oligosaccharides play an essential role in osmotic stress [30,31].

Polyamines participate in abiotic stress, and the accumulation of polyamines enhances resistance to abiotic stress in plants [32]. Spermidine can improve photosynthetic capacity and participate in hormone signal transmission under stress [33]. Overexpression of *speE* increased spermidine accumulation and enhanced tolerance to multiple environmental stresses in Arabidopsis [34]. In addition, the increased activity of *PAOs* in the backconversion pathway that catalyzes spermine and spermidine to putrescine is also involved in abiotic stress [35]. *SpeD*, *SpeE* and *PAO2*, *3*, *4* and *ALDH7A1* (two genes) expression was up-regulated in ‘Qinghai’ after 2 h and 50 h of osmotic stress, indicating that polyamine biosynthesis and metabolism were involved in the response to osmotic stress, and polyamine accumulation was associated with the strong drought tolerance of ‘Qinghai’. GABA is also an important molecule and it participates in plant protection, promoting ethylene (ETH) production and affecting plant growth [36]. *ALDH7A1* expression was up-regulated, and amino acid content increased in the metabolic pathway in ‘Qinghai’ after osmotic stress, indicating that GABA may be also important for resisting osmotic stress in ‘Qinghai’.

Phenylalanine catalyzes the oxidation of glutamate–gamma–semialdehyde into glutamate with the reduction of NAD (+) into NADH, and is involved in osmotic regulation. Glutamate plays multiple roles in abiotic stresses, such as salt, cold, heat and osmotic [37,38]. The large number of genes in the arginine and proline metabolism pathways were up-regulated after osmotic stress in osmotic-tolerant plants [39]. P5CS and P5CR are key enzymes in the proline synthesis pathway, catalyzing proline synthesis from glutamate. These observations are consistent with our analysis. Thirty-one genes involved in amino acid biosynthesis and metabolism were up-regulated in leaves in response to osmotic stress, including P5CS and P5CR. It is likely that the levels of 18 amino acids increased after osmotic stress. Among them, Pro and Phe levels showed 20 and 33.5-fold increase after osmotic stress, indicating increased proline and phenylalanine content is important for enhancing osmotic tolerance in ‘Qinghai’.

ABA is a defensive phytohormone and regulates stomatal closure, gene expression and the accumulation of osmotic protectants [40]. β-carotene is the precursor of ABA biosynthesis. It is hydrolyzed to produce zeaxanthin catalyzed by CrtZ, while zeaxanthin is further oxidized to produce violaxanthin. Violaxanthin is converted to ABA via several steps catalyzed by NCED, ABA2 and AAO. *CrtZ*, *ABA2*, *NCED*, *AAO3* and *CYP707A* expression was up-regulated in ‘Qinghai’ after osmotic stress. The accumulated ABA activates downstream signaling components to mediate signal cross-talking with other pathways [41]. The abscisic acid receptors PYR/PYLs are a positive factor combining with ABA to inhibit PP2C by phosphorylation in ABA signal transduction. The phosphorylated PP2C release the SnRK2, while SnRK2 further regulate ABF by phosphorylation to induce related genes expression. The expression of most ZmPP2Cs were dramatically induced by multiple stresses in Maize (drought, salt, and ABA) [42]. Overexpression of ABF could improve osmotic tolerance in plants [43]. In our study, the expression of *PP2C*, *SnRK2s* and *ABF* were up-regulated, while four *PYL* genes were down-regulated after osmotic stress. There may be a complex balancing mechanism of osmotic stress in ‘Qinghai’.

Gibberellins are an antagonist of ABA in the regulation of drought tolerance [44]. Gibberellins (GA) promote plant growth, but negatively regulate drought tolerance [45]. GA2ox catalyzes the oxidation of Gas, and positively regulates drought tolerance [46]. Gibberellin receptor gene GID1 was reported to regulate stomatal development. The *gid1* mutant showed impaired biosynthesis of endogenous GA under drought stress [47]. Transcription factor *PIF4* was reported to regulate auxin biosynthesis and is involved in stress response [48]. Consistent with these observations, *GA2ox*, *GID1* and *PIF4* expression was also up-regulated to synergistically regulate plant development and response to osmotic stress.

## 4. Materials and Methods

### 4.1. Plant Growth and Osmotic Stress

The Kentucky bluegrass ‘Qinghai’ (*Poa pratensis* cv. Qinghai) were seeded in plastic pots containing a mixture of peat and vermiculite and grown in greenhouse at 25 °C under nature light. Two-month-old seedlings were washed carefully and incubated in 1/2 Hoagland nutrient solution for one week before osmotic treatment. The seedlings were transferred to 1/2 Hoagland nutrient solution containing 25% (*v*/*v*) PEG-6000, while 1/2 Hoagland nutrient solution was used as the control. Leaves and roots were harvested after 0 h, 2 h and 50 h of stress treatment. RNA was isolated for transcriptome sequencing analysis. Free amino acids and soluble sugars were measured after 50 h of stress treatment.

### 4.2. RNA Isolation and RNA-Seq Analysis

Total RNA was extracted from 0.5 g roots and leaves using IZOL reagent (Invitrogen, Carlsbad, CA, USA) according to the manufacturer’s instructions [26], three RNA samples at each time point were used to construct a cDNA library of repeats using Gene Denovo Biotechnology Co. (Guangzhou, China) as described by Li et al. (2022) [26] for sequencing using Illumina HiSeq TM 2500. The RNA-seq data were deposited in the sequence read archive (SRA) of the NCBI database (accession number: PRJNA1025311). The FASTQ formatted raw sequence reads were pre-processed through in-house perl scripts by removing reads containing an adapter and those with more than 5% “N” base, and low-quality reads (length > 50% of the bases at *p*-value ≤ 5) were removed to obtain high-quality clean data. The cleaned reads were performed de novo transcriptome assembly using Trinity (v2.90) with default settings [49].

All assembled unigenes were subjected to alignment and annotation using the following databases: Non-Redundancy Protein (NR) database (http://www.ncbi.nlm.nih.gov/, accessed on 2 June 2021), Swiss-Prot database (http://www.expasy.ch/sprot/, accessed on 2 June 2021), Pfam database (http://pfam.xfam.org/, accessed on 2 June 2021), Cluster of Orthologous Groups of proteins (COG) database (http://www.ncbi.nlm.nih.gov/COG, accessed on 2 June 2021), Gene Ontology (GO) database (http://www.geneontology.org, accessed on 2 June 2021) and the Kyoto Encyclopedia of Genes and Genomes (KEGG) database (http://www.genome.jp/kegg/, accessed on 11 November 2023) [50]. The BLASTx algorithm (v2.2.28+) was employed with an E-value threshold of ≤10^−5^ for database searches. The best BLAST hit was used to determine the sequence orientation of the unigenes

### 4.3. In-Depth Analysis of Differential Gene Expression

The reference sequence used for mapping the clean reads of each sample was generated from transcriptome sequences assembled using Trinity. Mapping was performed using RSEM (v1.3.1) (http://deweylab.biostat.wisc.edu/rsem/, accessed on 2 June 2021) (bowtie 2, the parameter is mismatch 0). The expression levels of individual unigenes were quantified using the metric of transcripts per kilobase of exon model per million mapped reads (TPM). Differential gene expression analysis was conducted using DESeq (v1.20.0) software; the unigenes were determined as differentially expressed genes (DEGs) with false discovery rate (FDR) ≤ 0.05 or absolute log_2_ (foldchange (FC)) value ≥ 1, FC = FPKM (treat)/FPKM (control), FC > 2 or FC < 0.5. Enrichment analysis for differentially expressed genes in KEGG pathways and GO terms was performed using the R package “ClusterProfile” [51], with a *q*-value ≤ 0.05 to identify significantly enriched GO terms and KEGG pathways.

### 4.4. Trend Analysis

To understand the expression patterns of DEGs, trend analysis was used to cluster genes with similar expression patterns in different time samples. Clustering of the DEGs was performed by using version 1.3.13 of Short Time-series Expression Miner (STEM) software [52], and clustered profiles were considered significant when *p* values were ≤0.05.

### 4.5. Measurements of Free Amino Acids

Fresh leaves (0.2 g) were powdered and soaked in 3 mL of 20 mM hydrochloric acid for measurements of free amino acids as previously described [53]. The mixture experienced 30 min of ultrasound vibration and was centrifuged at 12,000 rpm for 10 min. Then, 1 mL of the supernatant was mixed with 1 mL of 5% sulfosalicylic acid and placed at room temperature for 1 h and centrifuged at 12,000 rpm for 10 min. The supernatant was filtered with a 0.22 µm membrane and the free amino acids were measured using automatic amino acid analyzer Hitachi L-8900.

### 4.6. Measurement of Soluble Sugars

The extraction of soluble sugars was based on a previously method [54]. Fresh leaves (0.5 g) were harvested and dried at 80 °C in a baking oven. The dried leaves were powdered and soaked in 5 mL of 80% (*v/v*) ethanol in a water bath at 80 °C for 30 min. The mixture was cooled at room temperature and centrifuged at 14,000 rpm for 20 min. The supernatant was combined after 3 repeated extractions and evaporated in a water bath at 80 °C. Then, the samples were dissolved in 1 mL of ultrapure water and filtered with a 0.22 μm membrane before testing. The extracts were separated on acetonitrile-water and water with a Carbohydrate column (4.6 mm × 250 mm, 5-Micron; Agilent Technologies Inc., Santa Clara, CA, USA) and a Hi-Plex Ca column (4 mm × 250 mm, 8 µm; Agilent Technologies Inc.), respectively, and were analyzed using the Agilent Technologies 1260 Infinity II with RID detector. Sucrose, raffinose, trehalose, glucose, galactinol and fructose concentrations were calculated based on the standard curve for each sugar and calibrated with the recovery of the whole analysis procedure.

### 4.7. Quantitative Real-Time PCR (qRT-PCR) Analysis

Total RNA was extracted from the roots and leaves of the Kentucky bluegrass using TRIZOL reagent (Invitrogen, CA, USA) according to the manufacturer’s instructions. The cDNA was synthesized using the PrimeScript™ II 1st Strand cDNA Synthesis Kit (Solarbio, Beijing, China) according to the manufacturer’s instructions. The qRT-PCR was performed using SYBR^®^ Premix Ex Taq^TM^ II kit (Takara Biomedical Technology, Dalian, China) and a Light Cycl^®^96 Real-Time PCR system (Roche Life Science, Shanghai, China) according to the manufacturer’s instructions. The reaction conditions were 94 °C for 5 min, followed by 40 cycles of 95 °C for 15 s and 60 °C for 1 min. The *PpActin* gene was used as the internal control. The relative expression level of gene was calculated using the 2^−ΔΔCt^ method [55]. The specific primers were listed in Supplementary Table S4. Three independent technical repeats and three biological replicates were performed.

## 5. Conclusions

Transcriptome analysis of Kentucky bluegrass cultivar ‘Qinghai’ showed that the majority of genes were up-regulated in leaves but down-regulated in roots in response to osmotic stress. The genes involved in stress-related pathway, such as carbohydrate metabolism, polyamine and amino acid metabolism and plant hormone signaling pathways were altered. The levels of sucrose, trehalose and raffinose, as well as amino acids, such as Glu, Val, Met and Pro, were increased after osmotic stress. Overall, our study contributes to a systematic understanding of changes in DEGs and critical metabolism in ‘Qinghai’ after osmotic stress. This study provides a theoretical basis for studying drought mechanisms in ‘Qinghai’.

## Figures and Tables

**Table 1:** Amino acids and their derivative levels in leaves in response to osmotic stress.

Amino Acid and Derivative	Control (μg g^−1^ DW)	Osmotic Stress (μg g^−1^ DW)
Glutamate (Glu)	642.88 ± 77.95	1142.38 ± 15.92 **
Ornithine (Orn)	0.17 ± 0.016	0.74 ± 0.34
Glycine (Gly)	0.57 ± 0.06	1.98 ± 0.47 *
Methionine (Met)	0.35 ± 0.03	2.20 ± 0.13 **
Threonine (Thr)	8.99 ± 2.47	26.50 ± 6.39 *
Asparagine (Asp)	9.61 ± 0.76	33.23 ± 10.70
Serine (Ser)	9.06 ± 2.49	40.53 ± 17.01
Alanine (Ala)	4.33 ± 0.35	21.29 ± 5.68 *
Arginine (Arg)	0.54 ± 0.19	6.22 ± 2.19 *
Lysine (Lys)	0.52 ± 0.17	9.23 ± 3.77
Tyrosine (Tyr)	0.55 ± 0.37	9.92 ± 4.44
Histidine (His)	1.17 ± 0.16	15.54 ± 5.19 *
Tryptophan (Trp)	1.04 ± 0.78	15.25 ± 5.53 *
l-isoleucine (Ile)	1.14 ± 0.36	21.32 ± 5.00
Leucine (Leu)	0.34 ± 0.14	14.68 ± 6.41 *
Proline (Pro)	5.03	102.41 ± 40.42 *
Phenylalanine (Phe)	0.92 ± 0.39	30.21 ± 10.76 *
Valine (Val)	0.94 ± 0.40	33.48 ± 12.98 **
Cysteine (Cys)	1.27 ± 0.26	1.30 ± 0.42
Taurine (Tau)	0.01 ± 0.00	0.35 ± 0.29
α-aminobutyricacid (GABA)	1.09 ± 0.44	47.60 ± 17.95 *
Cysthionine (Cysthi)	0.08 ± 0.02	0.03 ± 0.00
α-aminoadipic acid (α-AAA)	1.40 ± 0.09	13.68 ± 4.43 *
β-aminoisobutyric acid (β-AiBA)	1.14 ± 0.99	1.81 ± 1.14
P-Serine (P-Ser)	0.99 ± 0.26	2.95 ± 0.21 *
β-Alanine (β-Ala)	0.18 ± 0.05	1.71 ± 0.60 *
1-methylhistidine (1-Mehis)	0.39 ± 0.06	3.66 ± 2.44
α-aminobutyricacid (α-ABA)	0.57 ± 0.06	4.62 ± 1.14 *
Total	696.27	1573.99

Means of three independent samples and standard errors are presented. The asterisks * and ** indicate significant difference at *p* < 0.05 and 0.01, respectively.

## Data Availability

Raw Illumina sequence data were deposited in the National Center for Biotechnology Information (NCBI) and be accessed in the sequence read archive (SRA) database (https://www.ncbi.nlm.nih.gov/sra, accessed on 7 October 2023). The accession number is PRJNA1025311 (https://www.ncbi.nlm.nih.gov/bioproject/PRJNA1025311, accessed on 7 October 2023), which includes 18 accession items (SAMN37714455-SAMN37714472). All data generated or analyzed during this study are included in this published article and it supplementary information files.

## Supplementary Materials

The following supporting information can be downloaded at: https://www.mdpi.com/article/10.3390/plants12233971/s1, Table S1. Data quality summary of samples. Table S2. Summary statistics of the common vetch transcriptome assemblies. Table S3. Number of functional comments. Table S4. Specific primers of quantitative real-time PCR (qRT-PCR). Figure S1. GO enrichment analysis of the up- and down-regulated DEGs in leaves and roots. Figure S2: Analysis of DEGs involved in signal transduction of plant hormones (IAA, CTK, ETH, BR, JA) in leaves in response to osmotic stress.
