# Supplementary material for: Transcriptome Analysis of Native Kentucky Bluegrass (Poa pratensis L.) in Response to Osmotic Stress
Source: Plants (Basel). 2023 Nov 25;12(23):3971. doi: 10.3390/plants12233971 (PMC10708062; doi:10.3390/plants12233971)
Supplement: Supplementary file 1 [file plants-12-03971-s001.zip › plants-2676920-supplementary.pdf]

Supplementary Information

Table S1. Data quality summary of samples

	Raw reads	Clean reads	Error (%)	Clean_bases	Q20	Q30
L0_1	25866131	25041924	0.02	7.51G	98.4	95.25
L0_2	23219099	22531956	0.02	6.76G	98.45	95.38
L0_3	23442426	22942083	0.02	6.88G	98.29	94.86
L2_1	23064899	22694089	0.02	6.81G	98.41	95.16
L2_2	23686522	23222260	0.02	6.97G	98.35	95.03
L2_3	20569478	20091708	0.02	6.03G	98.28	94.91
L50_1	23223593	22901918	0.02	6.87G	98.37	95.01
L50_2	22782252	22367453	0.02	6.71G	98.43	95.24
L50_3	20966916	20613887	0.02	6.18G	98.29	94.85
R0_1	21419350	20987087	0.02	6.30G	98.2	94.48
R0_2	22339635	21803945	0.02	6.54G	98.2	94.62
R0_3	21462951	21068240	0.02	6.32G	98.15	94.38
R2_1	21982104	21569433	0.02	6.47G	98.06	94.42
R2_2	21593969	20965483	0.02	6.29G	98.19	94.76
R2_3	22621121	22283473	0.02	6.69G	98.1	94.4
R50_1	22975970	21779479	0.03	6.53G	96.9	92.9
R50_2	23162466	22295946	0.02	6.69G	98.07	94.71
R50_3	23374197	22984339	0.02	6.90G	98.25	94.89
Total	407753079	398144703	-	119.45G	-	-

Raw reads: the number of reads in the original data. Clean Reads: the number of reads filtered from the original data. Error (%): rrror rate of sequencing. Clean bases: base number filtered from raw data. Q20: percentage of bases with Phred values greater than 20 in total bases. Q30: percentage of bases with Phred values greater than 30 in total bases.

Table S2. Summary statistics for the common vetch transcriptome assemblies

Length_interval	Transcripts	Unigenes
300bp-500bp	436314	273637
500bp-1kbp	377759	187379
1kb-2kbp	211158	82764
>2kbp	65613	25490
Total number	1090844	569270
Min_length	301	301
Mean_length	831	737
Median_length	592	515
Max_length	16653	16653
N50	1049	870
N90	398	371
Total_nucleotides	906582325	419561752

Length interval: the different length of transcriptome assemblies. Min length: the minimum length of transcriptome assemblies. Mean length: the average length of transcriptome assemblies. Median length: the median length of transcriptome assemblies. Max length: the maximum length of transcriptome assemblies. N50: the transcriptome assemblies are sorted from long to short in length and accumulated to more than 50% of total length. N90: the transcriptome assemblies are sorted from long to short in length and accumulated to more than 90% of total length.

Table S3. Number of functional comments

Database	Number of Unigenes	Percentage (%)
Annotated in Nr	271475	47.68
Annotated in Nt	116438	20.45
Annotated in KO	121782	21.39
Annotated in SwissProt	204584	35.93
Annotated in PFAM	248701	43.68
Annotated in GO	248683	43.68
Annotated in KOG	103921	18.25
Annotated in all Databases	23636	4.15
Annotated in at least one Database	365785	64.25
Total Unigenes	569270	100

Nr: NCBI non-redundant protein sequences. Nt: NCBI nucleotide sequences. KO: Kyoto Encyclopedia of Genes and Genomes. SwissProt: a manually annotated and reviewed protein sequence database. PFAM: protein family. GO: gene ontology. KOG: euKaryotic Ortholog Groups.

Table S4. Specific primers of quantitative real-time PCR (qRT-PCR)

Gene ID	Sequense	T_m	Primer name
Cluster-293458.138681	GACATTGCCAGCTGACAACC	59.8	GAPA-F
Cluster-293458.138681	GGAGGTCATGCATTGCAACG	60.2	GAPA-R
Cluster-293458.138507	CCAAGAGGTATCGGTGCTGG	60.2	PRK-F
Cluster-293458.138507	CTCGAGAGGTTGCTCAGGTG	60.1	PRK-R
Cluster-293458.138203	CTGAACTCCAGGCAGGGAAC	60.3	rbcS-F
Cluster-293458.138203	GCATGCAGGTTTGGCCAATT	60.3	rbcs-R
Cluster-293458.141288	TGACAGTCAGGCCAAATGCT	59.9	SUSase-F
Cluster-293458.141288	CCTGGAGTTTGCCGTGTACT	60	SUSase-R
Cluster-293458.132279	GAGAGCGAGGACAGGTTCAG	59.8	ABF-F
Cluster-293458.132279	TTCGGACAAGGAGTTGGTGG	59.9	ABF-R
Cluster-293458.142366	GAAGCAAAGCGATCCCCTCT	60.1	amyB-F
Cluster-293458.142366	TTCTGTGTGATCGGCTCGAG	59.8	amyB-R
Cluster-293458.144677	ACTCCTCCTTGCAAGAACGG	60	HK-F
Cluster-293458.144677	TTCCGAGCGAAACAGAGCAT	60	HK-R
Cluster-293458.117885	TTCGACCCGGACGTAAAAGC	60.7	RAFS-F
Cluster-293458.117885	GGACAGGGTCGTCATCCAAG	60.1	RAFS-R
Cluster-293458.135562	TCCTGGGGTCACTCTGTCTC	60.3	INV-F
Cluster-293458.135562	TTGGCATCGAATGGGGAGTC	60.1	INV-R
Cluster-293458.140936	TGTCTGTCCACTACCGCAAC	60	otsB-F
Cluster-293458.140936	TCAGCTGCGTCCATAGAAGC	60.2	otsB-R

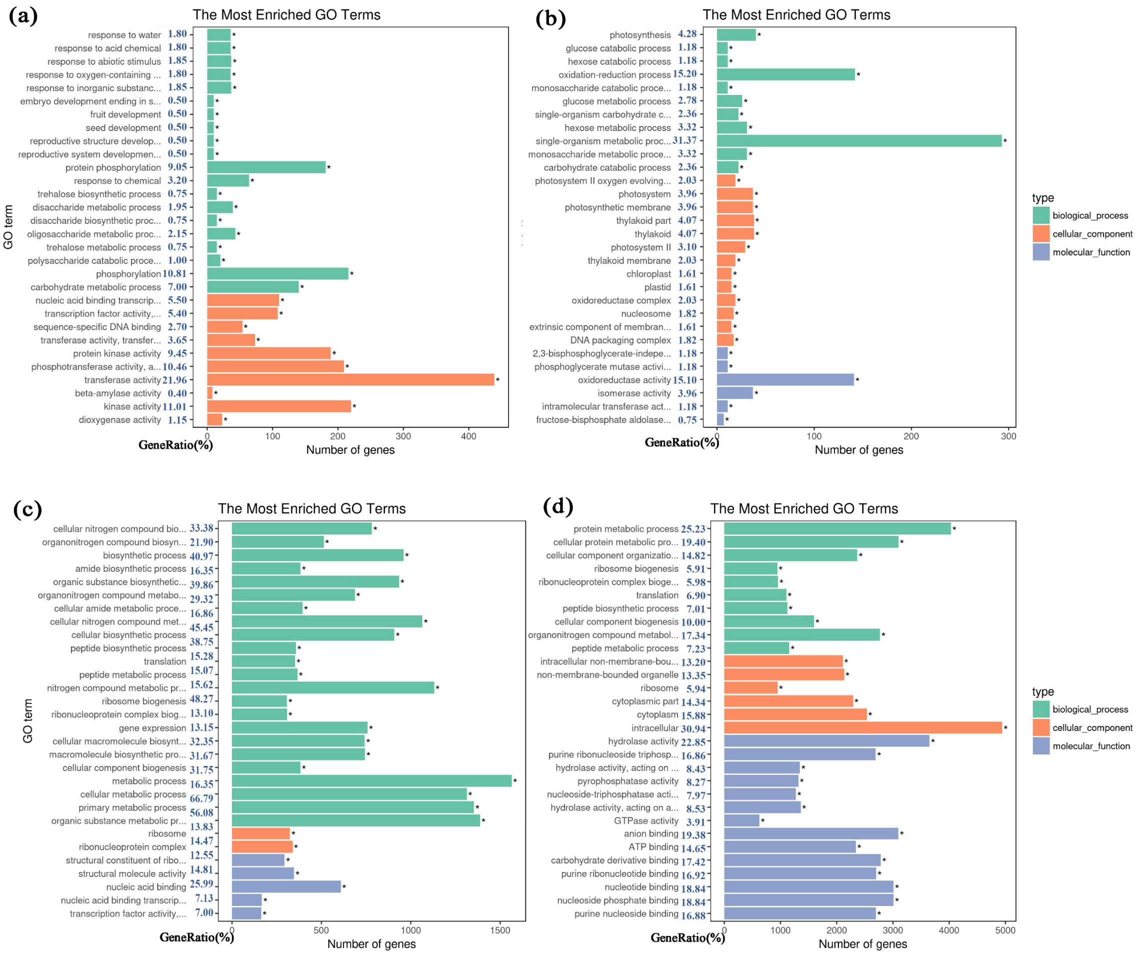

Figure S1. GO enrichment analysis of the up- and down-regulated DEGs in leaves and roots. (**a**) The most enriched GO terms of the up-regulated DEG in leaves; (**b**) the most enriched GO terms of the down-regulated DEGs in leaves; (**c**) the most enriched GO terms of the up-regulated DEG in roots; (**d**) the most enriched GO terms of the down-regulated DEGs in roots. Single (*P < 0.05) and two asterisks (**P < 0.01) represent significant differences in the student's t-test between the different treatments.

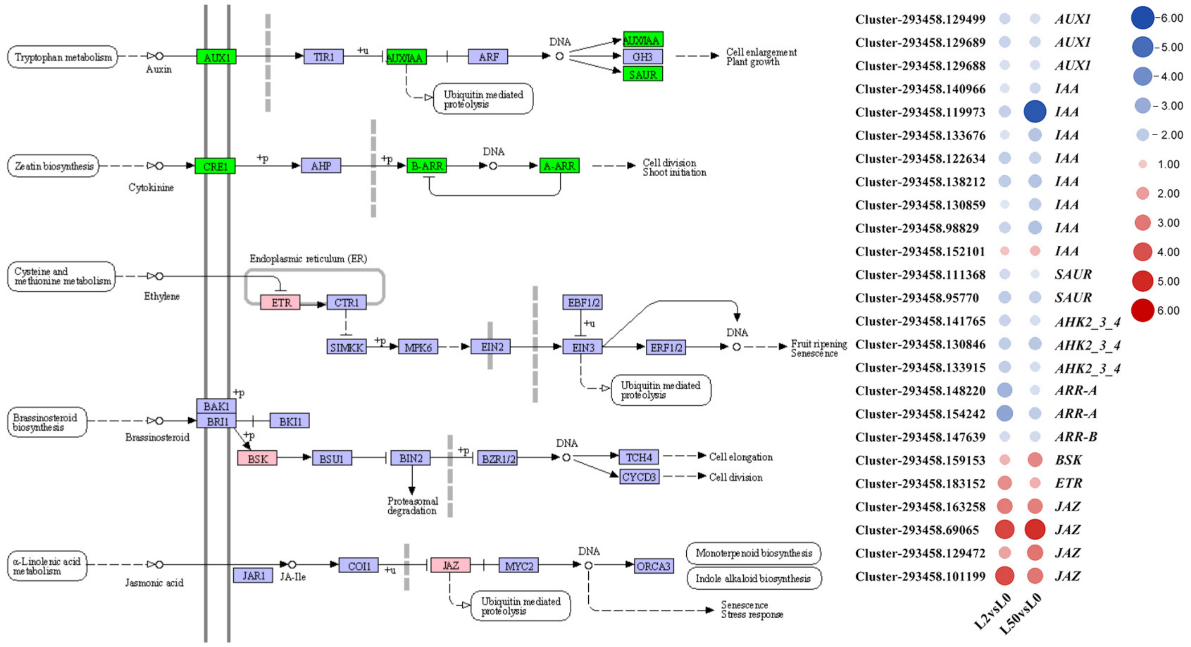

Figure S2. Analysis of DEGs involved in signal transduction of plant hormones (IAA, CTK, ETH, BR, JA) in leaves in response to osmotic stress. Schemes were adapted from KEGG (ko04075). Shapes and arrows follow the KEGG representation standards (https://www.kegg.jp/kegg/) (assessed on 11th November 2023), except for color codes. The green rectangles represent down-regulated genes, while the pink rectangles represent up-regulated genes. The color spectrum of heat map ranging from blue to red represents log_2FC values from low to high.
